# Concomitant Extramedullary Plasmacytoma in the Oropharynx and Hypopharyngeal Squamous Cell Carcinoma

**DOI:** 10.1155/2018/1463218

**Published:** 2018-07-31

**Authors:** Hiroki Sato, Shoko Yoshimasu, Isaku Okamoto, Akira Shimizu, Yasuaki Katsube, Hideki Tanaka, Kiyoaki Tsukahara

**Affiliations:** Department of Otorhinolaryngology, Head and Neck Surgery, Tokyo Medical University, Tokyo, Japan

## Abstract

We report a rare case of hypopharyngeal squamous cell carcinoma occurring synchronously with extramedullary plasmacytoma (EPM) of the oropharynx in which radiotherapy was used as the curative treatment. A 73-year-old man presented with a sore throat that had persisted for 6 months. Examination revealed a superficial, smooth tumorous lesion at the base of his tongue with a red hue in the oropharynx. In addition, a protruding tumor was observed on the mucosal surface in the right piriform recess of the hypopharynx, and computed tomography revealed thickening of the pharyngeal wall at the right tongue base and in the right piriform recess of the hypopharynx. Because no definitive diagnosis could be reached for the lesion at the base of the tongue, the entire tongue-base tumor was resected by transoral surgery under endoscopy. Proliferation of plasma cells in the tumor was detected, and a bone marrow puncture test ruled out multiple myeloma leading to a definitive diagnosis of Stage I (cT1N0M0) squamous cell carcinoma in the right piriform recess of the hypopharynx and primary extramedullary plasmacytoma in the oropharynx. Radiotherapy was selected for curative treatment with a complete response for both cancers. No recurrences have been observed as of 12 months postoperatively.

## 1. Introduction

Extramedullary plasmacytoma (EPM) is a tumor that originates from plasma cells. EPM develops in soft tissues other than bone marrow, frequently in the head and neck area. A large number of the malignant tumors developing in the head and neck area are squamous cell carcinomas, and such carcinomas often occur together with other malignant tumors, either synchronously or metachronously [1, 2]. However, head and neck squamous cell carcinoma occurring synchronously with EPM is extremely rare. A search in PubMed revealed only 3 cases of EPM coexisting with head and neck squamous cell carcinoma: a case of maxillary sinus EPM together with lingual squamous cell carcinoma; a case of laryngeal EPM together with laryngeal cancer; and a case of nasopharyngeal EPM together with nasopharyngeal cancer [3–5]. The present case of EPM coexisting with hypopharyngeal carcinoma is the first such case to be reported. Generally, surgery or radiotherapy is considered curative treatment for both EPM and head and neck squamous cell carcinomas. When EPM and head and neck squamous cell carcinomas are present together, adequate consideration of the treatment modality is necessary. We report a case of hypopharyngeal squamous cell carcinoma occurring synchronously with EPM of the oropharynx in which radiotherapy was used as the curative treatment.

## 2. Case Presentation

The patient was a 73-year-old man. He was examined at our hospital for a sore throat that had persisted for 6 months. He had a drinking habit of one 500 ml bottle of beer daily and no history of smoking. Diabetes, hypertension, and hyperlipidemia were noted in his previous medical history.

**Figure 1:**
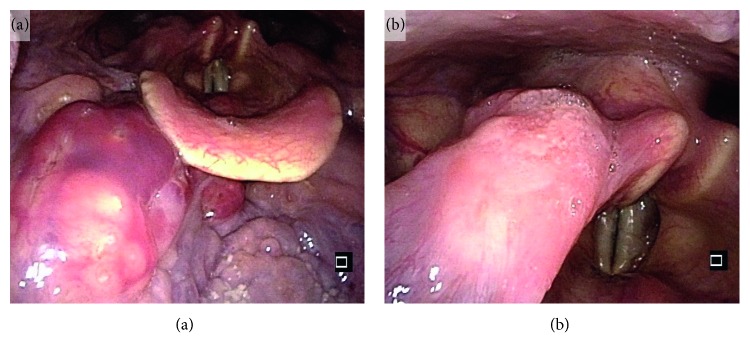
Pharyngolaryngoscopy. A superficial, smooth tumorous lesion with red color is observed (a). A protruding lesion with atypical blood vessel formation in the mucosa surface is also observed (b).

**Figure 2:**
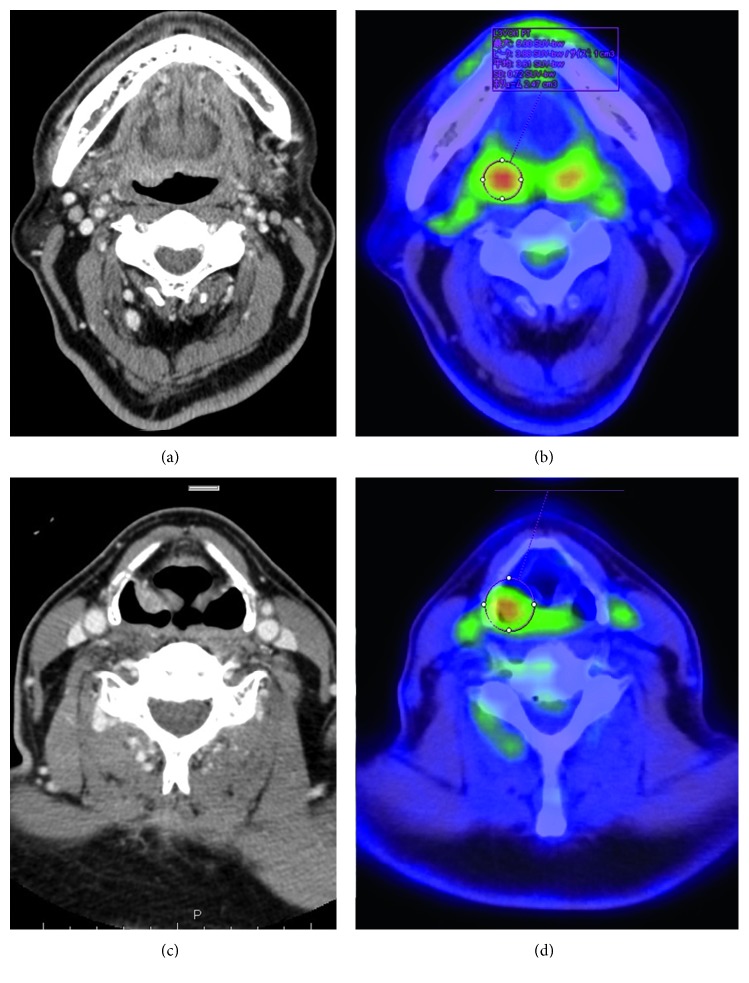
Contrast-enhanced CT and FDG PET/CT. Thickening of the pharyngeal wall with irregular contrast enhancement is observed on the right side of the tongue base and in the right piriform recess. SUVmax is 2.0 on the right side of the tongue base and 4.2 in the right piriform recess.

Pharyngolaryngoscopy revealed a superficial, smooth tumorous lesion with a red hue in the oropharynx at the base of the tongue. In addition, a protruding tumor with atypical blood vessel formation was observed on the mucosal surface in the right piriform recess of the hypopharynx (Figure 1). On contrast-enhanced CT, thickening of the pharyngeal wall showing irregular contrast enhancement was observed at the right tongue base and in the right piriform recess of the hypopharynx (Figure 2). No swelling of neck lymph nodes was observed. On fluorodeoxyglucose positron emission tomography/computed tomography (FDG PET/CT), accumulation was observed for maximum standardized uptake values (SUVmax) of 2.0 and 4.2 in the lesions of the oropharynx and hypopharynx, respectively (Figure 2). No accumulation was observed in neck lymph nodes or other parts.

**Figure 3:**
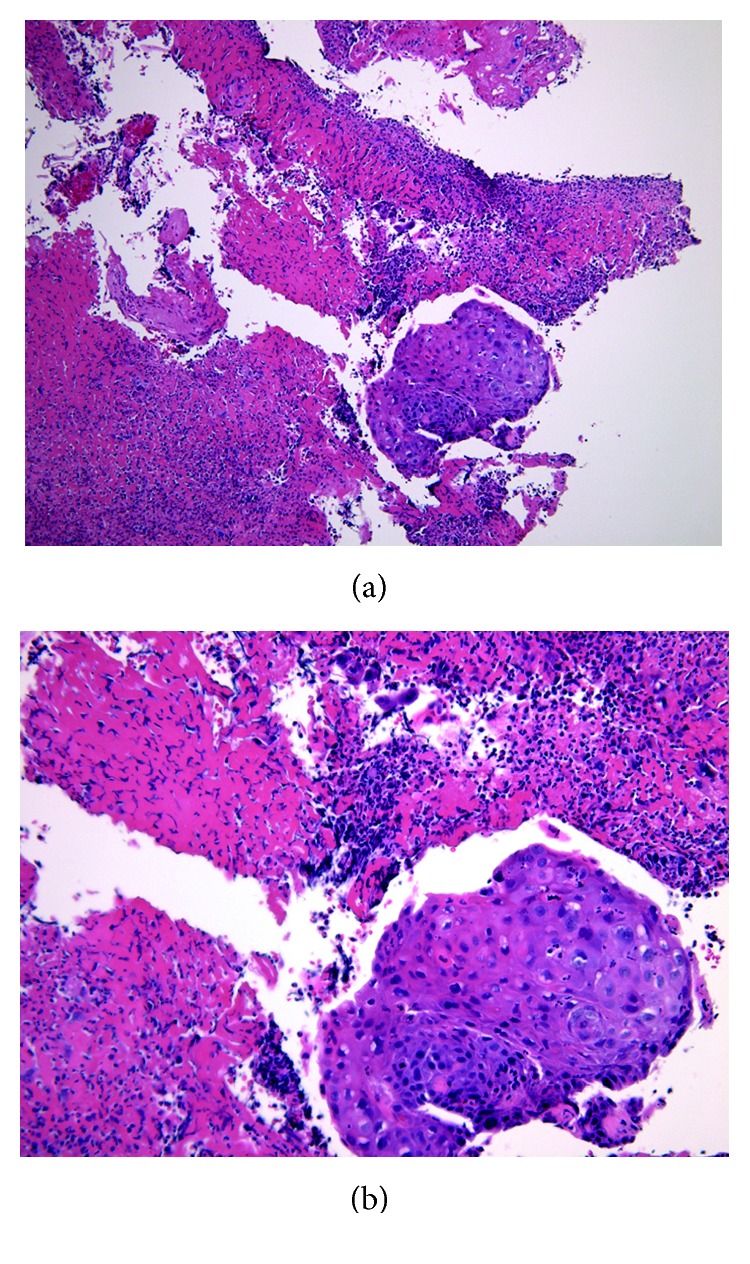
Histopathological findings for hypopharyngeal tumor biopsy. Features observed include increased nuclear chromatin, disparity between large and small nuclei, and signs of mitosis. These findings are indicative of squamous cell carcinoma.

**Figure 4:**
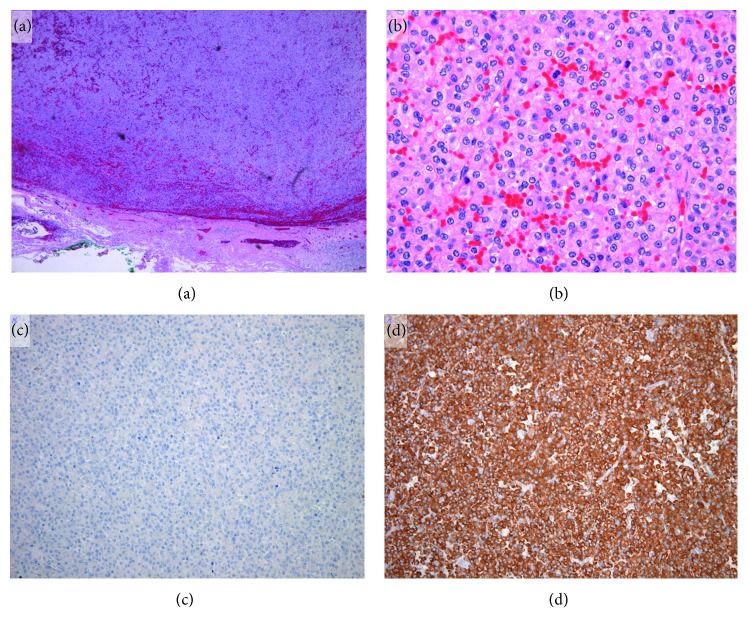
Histopathological findings for transorally resected nasopharyngeal tumor. HE staining: low magnification (a) and high magnification (b). Proliferation of plasma cells and unevenly distributed nuclei are observed. Immunostaining shows negative results for *κ* (c) and positive results for *λ* (d).

**Figure 5:**
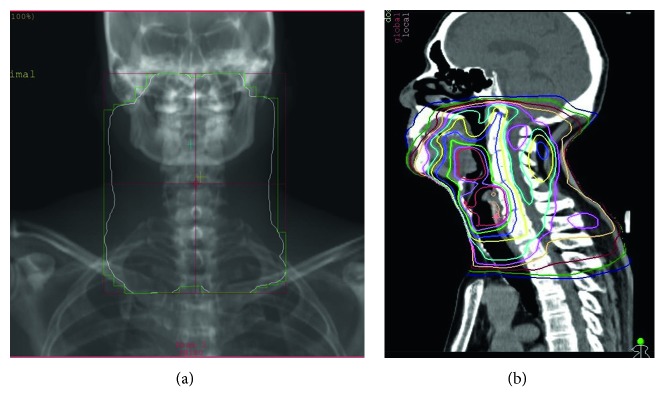
Distribution map of radiotherapy.

Based on tissue biopsies, the histopathological diagnosis for the lesion in the right piriform recess of the hypopharynx was squamous cell carcinoma (Figure 3). While the lesion at the base of the tongue was suspected to represent malignant lymphoma on histopathological examination, no definitive diagnosis could be reached. To achieve a definitive diagnosis, the entire tongue-base tumor was resected by transoral surgery under endoscopy. Subsequent histopathological examination revealed proliferation of plasma cells in the tumor, and immunostaining findings were as follows: *κ*(−), *λ*(+), CD3(−), CD20(−), CD138(−), CD79a(+), and MUM-1(+) (Figure 4). A bone marrow puncture test ruled out multiple myeloma. Based on the above, a definitive diagnosis of Stage I (cT1N0M0) squamous cell carcinoma in the right piriform recess of the hypopharynx and primary extramedullary plasmacytoma in the oropharynx was made. In the treatment plan, radiotherapy was selected for curative treatment. After irradiating the whole neck with 40 Gy in 20 fractions, the irradiation field was reduced to target the tumor in the hypopharynx only, and additional radiotherapy comprising 30 Gy in 15 fractions was conducted (Figure 5). Treatment outcome was complete response for both cancers, and no recurrences have been observed as of 12 months postoperatively.

## 3. Discussion

Locations where other cancers frequently occur together with head and neck squamous cell carcinomas are the esophagus, head and neck, stomach, and lungs. In 20% of esophageal cancer patients, other malignancies arise in the upper aerodigestive tract, and in 10%, they occur in the head and neck area [6, 7]. Synchronously occurring squamous cell carcinomas are thus generally suspected when multiple cancers are identified in the head and neck region. However, while accounting for just 3% of plasmacytomas as an extremely rare type of cancer, around 80% of EPMs develop in the head and neck region [8]. They mostly occur in the oral cavity, nasal cavity, pharynx, and larynx, so the frequency is higher in the upper respiratory tract [8]. Occurrence rates are 40% for the nasal cavity, 20% for the nasopharynx, and 18% for the oropharynx [9].

As stated in Introduction, among head and neck cancers, reported cases of concomitant squamous cell carcinoma and EPM are extremely rare, and this represents the first report of synchronous EPM and hypopharyngeal carcinoma. The mechanisms underlying joint occurrence of EPM and squamous cell carcinomas in the head and neck region have been unknown in all cases reported to date [3–5]. This is because the risk factors for both pathologies differ. When dual malignancies are observed in the head and neck area, EPM should be considered as a candidate for differential diagnosis, despite its rarity.

The National Comprehensive Cancer Network (NCCN) guidelines recommend radiotherapy or surgery for Stage I hypopharyngeal cancer [10]. Transoral resection is also increasingly being selected for patients with Stage I or II hypopharyngeal cancer. However, radiotherapy alone is frequently used for EPM because of its favorable radiosensitivity. Skóra et al. reported high response rates for radiotherapy alone in 17 cases of primary EPM in the head and neck, with 75% achieving complete response and 25% achieving partial response [11]. In the present case, we resected the EPM for biopsy purposes, and as a safety margin was not obtained at resection, we considered radiotherapy as warranted. Also, as the hypopharyngeal cancer was able to be treated under the same irradiation field, the hypopharyngeal carcinoma was also treated by radiotherapy.

EPM is an extremely rare disease that occurs mostly in the head and neck area, making this an important differential diagnosis for head and neck malignancies. Although no definitive diagnosis could be determined for the primary tumor in the oropharynx in the present case through the initial biopsy, complete resection for biopsy purposes enabled definitive diagnosis. As EPM may be locally recurrent or develop into multiple myeloma after treatment, pretreatment diagnosis is crucial. The follow-up protocol is that local recurrence, metachronous head and neck carcinoma, locoregional lymph node metastasis, and distant metastasis are assessed every 2–3 months by performing fiberscopy, CT, or cervical ultrasonography. In addition, metachronous esophageal and gastric carcinoma is assessed once a year by performing esophagogastroduodenoscopy. The screening for recurrence of the EPM is done using serum-free light chains every 6 months. After treatment, not only recurrence of the EPM but also of squamous cell carcinomas has to be kept in mind, as the latter frequently recur metachronously, necessitating rigorous follow-up.
